# Next Steps for Medical Specialist Enterprises in the Netherlands: Building Strong Clinical Governance and Leadership: Comment on "Alignment in the Hospital-Physician Relationship: A Qualitative Multiple Case Study of Medical Specialist Enterprises in the Netherlands"

**DOI:** 10.34172/ijhpm.8639

**Published:** 2024-09-09

**Authors:** Robin D.C. Gauld

**Affiliations:** Centre for Health Systems and Technology, University of Otago, Dunedin, New Zealand.

**Keywords:** The Netherlands, Medical Specialist Enterprises, New Zealand, Clinical Governance, Integrated Care, Primary Care

## Abstract

This commentary article responds to the research into development of medical specialist enterprises (MSEs) in the Netherlands conducted by Ubels and van Raaij. The MSEs are a relatively new phenomenon in the Netherlands and similar conceptually to medically-led developments in other health systems. With the foundation for medical specialist organisation in place this provides several opportunities for further development. This commentary considers these opportunities, drawing from the example of New Zealand. This is because New Zealand has had considerable experience with clinically-led organisation which provides useful lessons for the MSEs. The lessons include building strong clinical governance with a focus on collaboration with other health professionals and management, working with primary care to support community service delivery, building integrated care, developing whole of system planning and service delivery approaches and population health management.

 The Netherlands Medical Specialist Enterprises (MSEs) described in the article by Ubels and van Raaij are an interesting development in the global context of contractual arrangements for medical specialists working in hospitals.^1^ In common with many other parts of the world, the Netherlands specialists function in something of an open marketplace where services suppliers (ie, medical specialists) often work with multiple different customers (ie, hospitals and patients). Any marketplace naturally leads to different terms of engagement and contractual arrangements, and different responses to changing regulatory conditions. In the Netherlands, specialists are able to work either as direct hospital employees or as individual self-employed contractors. The self-employed can work with several hospitals. As described by Ubels and van Raaij, there have been ongoing efforts and reforms with associated financial incentives in the Netherlands intended to align specialists more closely with individual hospitals. It is not uncommon for those procuring services of small or independent suppliers in a marketplace to pursue standard arrangements so they are able to better manage a system of suppliers, with the intent of improving quality and efficiency in the process and building economies of scale.^2^

 In healthcare, the policies put in place by the Dutch Healthcare Authority which led to the formation of the MSEs are not uncommon in that, as Ubels and van Raaij write, the MSEs were an unintended consequence of policy reforms. Similar policies have been pursued elsewhere with similarly unintended yet important results. A relevant example can be found in New Zealand where, in the early 1990s, the government sought to put in place a national contract for independent general practitioners (GPs) all of whom ran their own private businesses and worked on individual contracts with the government to provide patient services. While the New Zealand case involves GPs rather than hospital specialists, the circumstances and policy intent are not dissimilar to the MSE case. The New Zealand GPs, like many around the world, retained the right to directly charge patients for consultations while also receiving a substantial portion of their income from the government. The government sought to standardise the GP contract and also the expectations that would come with receiving taxpayer funding. This included some caveats around patient charges, some data collection requirements, and some quality and population management expectations.

 As with the MSEs, the New Zealand case led to the formation of what at the time was considered to be an important example of organised general practice with wide-ranging global lessons.^3-5^ GPs formed themselves, under their own leadership, into a series of “Independent Practitioner Associations” (IPAs). As Ubels and van Raaij note of the MSEs, the IPAs were an unintended policy consequence. It is for this reason, and conceptual similarities between two countries’ health systems,^6^ that New Zealand experience is relevant to further development of the Netherlands MSEs.

 The New Zealand IPAs became a force to be reckoned with. Initially, they functioned as mechanisms for managing the contractual relationships of GP members with the government, with an IPA representing a large number of GPs. The goal was to achieve a balance in the contractual relationship and negotiation process. Very quickly, however, the IPAs transitioned into an example of clinical governance and leadership. The GP leaders recognised that their groups provided the capacity to work collaboratively across the range of areas. This included data collection across a population, building information systems specifically designed to achieve this purpose. It included negotiating with government agencies to manage budgets for prescribing and ordering diagnostic tests. With prescribing, the goal was to use the best evidence to reduce variation in prescribing and also the costs of prescribed medicines with any financial surplus able to be reinvested in new clinical services as agreed to by the IPA; same with diagnostic tests. The GPs also moved into building evidence for best clinical practices and laid the foundations for clinical guidelines, again with a goal of building evidence-based medicine and reducing variation. It was a very important period and empowered GPs with a sense of focus and pride that organisation was leading to better practice and patient care, ability to invest in new initiatives and building capacity for continuous improvement. Very importantly, the GPs established a strong position in terms of influencing public policy, again with goals of supporting and developing primary care, best practice and patient outcomes.^5^

 The Netherlands MSEs appear to represent a first step in terms of what is possible with specialist organisation in the country. As the authors describe, these enterprises have developed in a range of different ways across the five case study sites. This is a result of different contractual arrangements. The authors note that, where it is the focus and promoted, the relational aspects of the contract and activities between the MSE and constituent hospital are laying a strong foundation for making important progress in various areas. The interviewees cited in the article importantly note that joint strategy is one of the results from the relational contract. If so, and perhaps informing any follow up work on MSEs that Ubels and van Raaij undertake, there are a series of important next steps that could be pursued by the MSEs and built into their strategies. The rest of this response article discusses these, each of which has been pursued, with strong medical involvement, in the New Zealand health system.

 First, and perhaps most importantly, is the potential for developing a system of clinical governance and leadership.^7^ With MSEs functioning as a basic organisational form and a will in some of the cases to be involved in strategy the obvious next step is building clinical governance and being explicit about this. There is a good evidence base which shows that strong clinical governance and leadership is the best mechanism for improving hospital performance as well as health professional engagement.^8-11^ Clinical governance in general involves healthcare professionals working in a partnership with one another with a focus on responsibility for driving quality improvement. This is as professionals are directly involved in the system of care delivery. It is easy for individual health professionals to abrogate such responsibility, especially when they are individual contractors or parttime employees. As members of an MSE and with the right strategy, involvement in clinical governance could become a core component of medical specialist activity and MSEs could be the vehicle for promoting and supporting clinical governance.

 It may be that an initial step for the MSEs would be to develop a system of physician-led clinical governance given that these are specialist professional organisations. Once established, other professionals such as nurses and allied providers could be incorporated. Ideally, a strong system of clinical governance is a partnership between the various different professionals all of whom are involved as part of a team providing care.^12^ As the relational MSE cases note, strategy is a joint goal of both the hospital and the MSE. A strong system of clinical governance also involves a working partnership and joined up system of governance where management and professionals work in an equal partnership with shared goals of continually improving the quality of care. The job of management is to support clinically-agreed, evidence-based decisions around care processes and quality improvement. A robust system of clinical governance would also involve professionals in all key hospital planning decisions, management and governance processes. This would include capital development projects, IT projects and other initiatives, for example. Thus, there is a potentially exciting and important future ahead for MSEs in contributing to all aspects of Netherlands hospital leadership.

 Second, with a strong clinical governance focus and structure and place, MSEs can transition into working on areas such as unwarranted clinical practice variation. This is a well-recognised phenomenon in most health systems, including the Netherlands.^13-15^ Physicians are best placed to lead on reducing unwarranted variation. The method for this is reliant on physicians working together with a goal of reducing variation. This entails access to and collection of data which can then be used by physicians so they can assist one another with reducing variation and ensuring that care and care processes including diagnostics, test ordering, prescribing and treatment protocols are as standardised as possible. There are good examples of hospitals, driven by physicians, that have made considerable progress on reducing unwarranted variation. One of the keys to this is doctor-to-doctor conversations, facilitated through a doctor-led organisational mechanism such as the MSE. MSEs could also lead on processes such as transparency around clinical quality performance, developing publicly-available indicators for this as has been done elsewhere. They could also lead on reducing unmet healthcare need, particularly for specialist care, evident and very troubling in most health systems.

 Third, MSEs have the foundations and potential to drive service integration. Again, this has been a goal in the Netherlands and in most developed world health systems and is challenging.^6^ At the centre of integration is the need for health professionals to be able to work collaboratively with one another in order to have conversations around patient journeys, resource allocation across the different levels of care—primary, secondary and tertiary—and inter professional partnerships. Very importantly, MSEs can play a role in working to support general practice and primary care. There would be opportunities, for example, for individual specialists to work through the MSE to hold outpatient clinics in GP and primary care settings; also, to assist GPs with better managing patients with secondary care needs in community settings, avoiding the need for hospitalisation. Where there are funding constraints to such working, MSEs would be well placed to facilitate conversations around what is needed to support integrated care initiatives.

 Fourth, there is a growing international focus on whole system planning and population health leadership.^16^ In practice, this means viewing the health system as a whole system rather than a series of parts bifurcated into primary and hospital care with separate funding and service delivery lines. Population health leadership refers to the demand for planning services in response to the needs of the population. This requires a focus on population data including demographics, health use patterns and health risks. The organisational model that MSEs represent provides an opportunity to analyse all of the resources available and being consumed within the whole system with a focus on planning where these resources might be allocated and most effectively applied. Such a planning focus can also assist with integrated care activities.^17^ The use of data is fundamental to providing leadership that will improve services configuration, quality and performance on behalf of the broader population. While the individual specialists who are members of MSEs may see themselves as individuals focused on best care for their individual patients, their original training in medicine oriented them toward doing their very best to improve health. It is in this spirit that MSEs can position themselves as leaders on whole system and population health planning.

## Conclusion

 As Ubels and van Raaij have found, is an exciting time in the Netherlands with the organisation of medical specialists through MSEs. The foundations have been laid for developing a series of important medically-led initiatives that will build a strong health system with goals of better service organisation, quality improvement and improved patient experience. This commentary article has described such initiatives implemented in New Zealand that could be adopted by MSEs. The challenge now is for the MSEs to pursue these activities.

## Ethical issues

 Not applicable.

## Conflict of interests

 Author declares that he has no conflict of interests.
